# Effects of Dietary Starch Structure on Growth Performance, Serum Glucose–Insulin Response, and Intestinal Health in Weaned Piglets

**DOI:** 10.3390/ani10030543

**Published:** 2020-03-24

**Authors:** Xiaoqian Gao, Bing Yu, Jie Yu, Xiangbing Mao, Zhiqing Huang, Yuheng Luo, Junqiu Luo, Ping Zheng, Jun He, Daiwen Chen

**Affiliations:** Key Laboratory of Animal Disease-Resistance Nutrition, Ministry of Education, Institute of Animal Nutrition, Sichuan Agricultural University, Chengdu 611130, China; gaoxiaoqian9579@163.com (X.G.); ybingtian@163.com (B.Y.); yujie@sicau.edu.cn (J.Y.); acatmxb2003@163.com (X.M.); zqhuang@sicau.edu.cn (Z.H.); luoluo212@126.com (Y.L.); junqluo2018@tom.com (J.L.); zpind05@163.com (P.Z.)

**Keywords:** dietary starch structure, growth performance, glucose–insulin response, intestinal health, weaned piglets

## Abstract

**Simple Summary:**

Carbohydrates are the most important energy source for monogastric animals, including humans, and dysregulation of carbohydrate metabolism has been associated with metabolic syndromes, such as type 2 diabetes mellitus (T2DM), hypertension, and obesity. Starch is the major carbohydrate source, consisting of amylose and amylopectin. This study investigated the effects of dietary starch structure on growth performance, serum glucose–insulin response, and intestinal health in weaned piglets, which may contribute to the principles of carbohydrate nutrition and facilitate the utilization of dietary starches.

**Abstract:**

To investigate the effects of dietary starch structure (amylose/amylopectin ratio, AR) on serum glucose absorption metabolism and intestinal health, a total of ninety weaned piglets (Duroc × (Yorkshire × Landrace)) were randomly assigned to 5 dietary treatments and fed with a diet containing different AR (2.90, 1.46, 0.68, 0.31, and 0.14). The trial lasted for 21 d. In this study, the growth performance was not affected by the dietary starch structure (*p* > 0.05). Diets with higher amylose ratios (i.e., AR 2.90 and 1.46) led to a significant reduction of the serum glucose concentration at 3 h post-prandium (*p* < 0.01), while high amylopectin diets (AR 0.31 and 0.14) significantly elevated The expression of gene s at this time point (*p* < 0.01). High amylopectin diets also increased the apparent digestibility of crude protein (CP), ether extract (EE), dry matter (DM), gross energy (GE), and crude ash (*p* < 0.001). Interestingly, diet rich in amylose (AR 2.90) significantly elevated the butyric acid content (*p* < 0.05) and decreased the pH value (*p* < 0.05) in the cecal digesta. In contrast, diet rich in amylopectin (i.e., AR 0.14) significantly elevated the total bacteria populations in the cecal digesta (*p* < 0.001). Moreover, a high amylopectin diet (AR 0.14) tended to elevate the mRNA level of fatty acid synthase (*FAS*, *p* = 0.083), but significantly decreased the mRNA level of sodium-dependent glucose transporter 1 (*SGLT1, p* < 0.05) in the duodenal and jejunal mucosa, respectively. These results suggested that blood glucose and insulin concentrations were improved in high AR diets, and the diet also helped to maintain the intestinal health.

## 1. Introduction

Diet plays an important role in the regulation and prevention of metabolic disorders. Therefore, the strategy of dietary therapy for metabolic diseases has gained attention. Starch is the main digestible carbohydrate component in diets for most monogastric mammals and is the major energy provider [1]. Diet starch is a mixture of amylose and amylopectin, and its nutritional value greatly depends on the ratio of amylose to amylopectin [2]. Resistant starch (RS) is also a part of starch that is not digested and absorbed in the small intestine. Generally, the RS level is associated with the ratio of amylose to amylopectin, whereby higher amylose content also leads to higher RS content [3]. Amylopectin is more easily digested than amylose, leading to rapid increases in blood glucose and insulin levels [4]. The starch chemical structure influences the rate of starch digestion. Amylose is primarily a linear polymer of α-1,4-linked D-glucose units, whereas amylopectin is highly branched, comprising a chain of α-1,4 and α-1,6-linked D-glucose units [5]. Amylose is more difficult to hydrolyze than amylopectin because amylose polymers have more intra-molecular hydrogen bonds and less surface area [6]. The α-1,4-linkages are hydrolyzed by amylase of the pancreas to produce maltose and maltotriose, which then become glucose that is absorbed in the blood [4]. In pigs, the long-term ingestion of a diet with low AR increases lipogenesis and correspondingly up-regulates expression of lipogenic genes, such as fatty acid synthase (*FAS*) and acetyl CoA carboxylase (*ACC*), in liver and adipose tissue. However, ingestion of a high AR diet may lead to mild blood glucose and insulin responses [7,8]. A previous study indicated that the amylose can be fermented by large bowel microbiota and then various short-chain fatty acids are produced (SCFAs), which are beneficial for the health of people suffering from obesity and type 2 diabetes mellitus T2DM [9]. However, it is not clear whether the amylose/amylopectin ratios could affect the glucose–insulin response and metabolism in piglets. In current studies, inconsistencies exist in the effects of amylose/amylopectin ratios on the growth performance of animals [10,11,12].

The objective of the present study was to determine the effects of dietary amylose/amylopectin ratios on growth performance, serum glucose–insulin response, and gut microbiota in weaned piglets, and provide a partial theoretical reference for starch nutrition and a long-term energy intake option for piglets and human.

## 2. Materials and Methods

The experimental protocol used in this study was approved by Sichuan Agricultural University Institutional Animal Care and Use Committee No. 69130079.

### 2.1. Starch

High amylose maize starch (25% amylopectin and 75% amylose) and high amylopectin maize starch (94% amylopectin and 6% amylose) were purchased from Shanghai Quanwang Biotechnology Co., Ltd. (Shanghai, China) and Shandong Fuyang Biological Starch Co., Ltd. (Dezhou, Shangdong, China), respectively.

### 2.2. Animal Management and Housing

The experiment was conducted on 90 healthy cross-bred (Duroc × (Yorkshire ×Landrace)) piglets weaned at 21 d of age, with an average initial body weight (BW) of 7.51 (SEM 0.05) kg. After 3 d of standard diet, piglets were randomly selected and allotted to 1 of 5 dietary treatments with different dietary amylose/amylopectin ratios (DAR) based on their body weight and litters. Each treatment was replicated, with 6 pens of 3 piglets per replicate pen. Replicates contained equal numbers of females and castrated males. Piglets within the same replicate were housed together in one pen (1.5 × 1.5 m^2^). Room temperature was maintained at 28 ± 1 °C and relative humidity was controlled at 65%–75% throughout the study. Piglets were fed the experimental diets ad libitum in a mash form for 3 weeks, and they had free access to water throughout the experiment. They were hand-fed four times/d (08.00, 12.00, 16.00 and 20.00 h) in bowl feeders to make sure fresh feed was available. All piglets were weighed at the beginning and the end of the experiment after 12 h of fasting. Feed intake was recorded every day to calculate average daily gain (ADG), average daily feed intake (ADFI), and the ratio of average daily feed intake to average daily gain (F/G) per pen.

### 2.3. Dietary Treatments

The experimental diets were formulated on the basis of nutrient requirements established by the National Research Council (NRC 2012, for 7–11 kg pigs (Table 1)) [13]. All diets were free of antibiotics. The 5 diet groups differed only in the starch component. The amylose/amylopectin ratios of the diet were 2.90 (50% amylose and 43.62% total starch), 1.46 (44% amylose, 6% amylopectin, and 45.57% total starch), 0.68 (32% amylose, 18% amylopectin, and 43.25% total starch), 0.31 (12% amylose, 38% amylopectin, and 43.76% total starch), and 0.14 (50% amylopectin and 46.06% total starch), respectively, which were obtained by adjusting the levels of high amylose maize starch and high amylopectin maize starch in the diet. 

### 2.4. Sample Collections

Experimental diets were sampled and stored at −20 °C for chemical analysis of dry matter (DM), ether extract (EE), crude protein (CP), crude ash, gross energy (GE), total starch, and ratio of amylose to amylopectin. From days 18 to 21, fresh fecal samples were collected and placed in individual plastic bags and then 10 mL of 5% H_2_SO_4_ solution was added to 100 g of each fresh fecal sample to fix excreta nitrogen [14].

After the trial, one medium-weight piglet from each pen was selected and venous blood samples were collected at 07.00. Piglets were fed at 08.00 and then postprandial blood samples were taken once an hour, from 09.00 to 13.50 h. Blood samples were centrifuged at 3000× *g* for 15 min at room temperature, and serum was subsequently separated and stored at −20 °C for further analysis. At the end of the study, the selected piglets were killed by intravenous injection of sodium pentobarbital (200 ml/kg, BW) [15]. The abdominal cavity was opened from the sternum to the pubis to expose the gastrointestinal tract, without damaging the wall of the digestive tract. The pH value of the cecal digesta was measured immediately with a pH meter (PHS-3C PH, Shanghai, China). The cecal digesta was collected into sterile containers and stored at −80 °C for measurement of microbial quantity (quantitative PCR) and volatile fatty acids (VFAs). The mucosa of the duodenum and jejunum was gently scraped with a glass slide and snap-frozen in liquid nitrogen, and then stored at −80 °C until further processing for relative mRNA expression analysis.

### 2.5. Apparent Digestibility of Nutrients and Starch 

Feces from the last 4 days of one medium-weight piglet from each pen were mixed thoroughly and dried at 65 °C for 72 h, after which they were ground to pass through a 40-mesh screen. The Apparent total tract digestibility (ATTD) was evaluated by using acid insoluble ash (AIA) as the digestibility indicator. The AIA in diet and fecal samples were determined by a method described by Mccarthy et al. [16] with modifications. The AIA content of the basal diet averaged 0.20 ± 0.002% DM. After AIA analysis, all feed and fecal samples were analyzed for DM (method 930.15, Association of Official Analytical Chemists (AOAC), 1995), ash (method 924.05, AOAC, 1995), EE (method 945.16, AOAC, 1995), CP (method 990.03, AOAC, 1995), and GE [17]. GE was determined using a specific adiabatic oxygen bomb calorimetry (Parr Instrument Co., Moline, IL, USA). The digestibility of chemical constituents was calculated using the following formula:

ATTD(%)=100− A1F2/A2F1×100

in which A1 = AIA content in diet, A2 = AIA content in feces, F1 = nutrient content in diet, and F2 = nutrient content of feces [18].

Total starch and AR were determined by assay kits (k-AMYL, Megazyme International Ireland Ltd., Wicklow, Ireland).

### 2.6. VFA Analysis

Cecal digesta samples were used to determine the concentration of VFA by gas chromatography according to Chen et al. [15]. Briefly, about 0.7 g sample was suspended in 1.5mL of ultrapure water in a centrifuge tube for 30 min. The entire sample was centrifuged at 15,000× *g* for 10 min. Then, 1 mL supernatant was transferred to a new sterile tube and then mixed with 0.2 mL 25% metaphosphoric acid and 23.3 μL 210 mmol/L crotonic acid simultaneously, then left at room temperature for 30 min. After 30 min, the sterile tubes were centrifuged at 15,000× *g* again for 10 min, then placed in sterile tubes in an ice bath for 30 min. Next, 500 μL supernatant was transferred to another sterile tube, mixed with 500 μL methanol, and homogenized for 10 min. After this, the mixture was centrifuged at 4 °C. Finally, the supernatant was injected into a gas chromatographic system (VARIAN CP-3800, Varian, Palo Alto, CA, America) to separate and quantify the VFA.

### 2.7. Serum Glucose and Insulin

The serum glucose concentration was measured by corresponding assay kits (Nanjing Jiancheng Institute of Bioengineering, Nanjing, China) according to the manufacturer’s instructions. The serum insulin concentration was measured by enzyme-linked immunosorbent assay (ELISA) kit (Jiangsu Meimian industrial Co., Ltd., Yancheng, China) according to the manufacturer’s instructions.

### 2.8. Total RNA Extraction and Real-Time Quantitative PCR

Total RNA was isolated from duodenal and jejunal mucous using TRIzol (Invitrogen, Carlsbad, CA, USA) and further purified by RNeasy Mini Kit (Qiagen, Valencia, CA, USA). All the procedures were carried out as per the manufacturer’s protocol. The concentration of RNA was determined using spectrophotometry based on the optical density (OD) ratio OD260/OD280, which ranged from 1.8 to 2.0 in all samples, while integrity was monitored using an Agilent 2100 bioanalyzer (Agilent Technologies, Santa Clara, CA, USA). The reverse transcription reaction was done using Real-time reagent kits with gDNA Eraser (TaKaRa), following the manufacturer’s instructions.

Real-time PCR primers were designed (Takara, Dalian) to assay four differentially expressed genes (Table 2). Here, *β*-Actin was used as the reference gene. Real-time PCR for four target genes and the house keeping gene was performed using Applied Biosystems (Foster City, CA, USA) Power SYBR Green PCR Master Mix in a Bio-Rad iCycler with minor modifications (Bio-Rad, Hercules, CA, USA). Fluorescein was added at a final concentration of 10 nM as the reference dye. Cycling conditions were as follows: 95 °C for 5 min, forty-five cycles of 95 °C for 30 s, appropriate annealing temperature (Table 2) for 30 s, 72 °C for 30 s, followed by 72 °C for 5 min, 95 °C for 1 min, 55 °C for 1 min, followed by a melt curve analysis of eighty cycles of 10 s at 55 °C, with a 0.5 °C increase every cycle.

### 2.9. DNA Extraction and Quantification of Intestinal Microflora

Microbial genomic DNA was isolated from the cecal digesta samples by using E.Z.N.A Stool DNA Kit (Omega Bio-Tek, Doraville, GA), according to the offered manual. Primers and probes (Table 3) for total bacteria, *E. coli*, *Lactobacillus*, *Bifidobacterium*, and *Bacillus* were obtained from the Qi et al. [19] and Fierer et al. [20], which were commercially synthesized by Invitrogen (Shanghai, China). Quantitative real-time PCR was performed with a CFX96 Real-Time PCR Detection System (Bio-Rad Laboratories, Inc., Hercules, CA). The total bacteria, *E. coli*, *Lactobacillus*, *Bifidobacterium*, and *Bacillus* were quantified by quantitative PCR with the method adapted from Diao et al. [21]. Copies per sample were calculated with the threshold cycle (CT) values and standard curve from the previous work [15]. Standard curves were generated using serial dilutions of the purified and quantified PCR products generated by standard PCR with the use of specific primers and genomic DNA from piglet digesta.

### 2.10. Statistical Analysis

Pen was the experimental unit for ADFI, ADG, and F: G. Gene expression data from replicate samples were averaged and analyzed using the Pfaffl [22] method to measure the difference of DAR. One medium-weight piglet from each pen was considered as an experimental unit for analyses of rest parameters. All data were subjected to one-way ANOVA for a completely randomized design using the general linear model (GLM) procedure of SPSS 20.0 (SPSS, Inc. Chicago, IL, USA). Statistical differences among the treatments were separated by Duncan’s multiple comparison test. Results were presented as means and standard errors (S.E.M). Statistical significance and a tendency towards difference were considered as *p* < 0.05 and *p* < 0.10, respectively. Regression analysis was used to estimate the linear and quadratic relation between dietary starch structure and dependent variables with the use of regression curve estimation.

## 3. Results

### 3.1. Growth Performance

The growth parameters are reported in Table 4. There were no significant differences (*p* > 0.05) in average daily gain or feed intake between the high AR and low AR groups during the 21 d experimental period.

### 3.2. Serum Glucose and Insulin

Fasting and postprandial glucose and insulin contents in serum are presented in Table 5 and Table 6, respectively. The dynamic time curves are shown in Figure S1. AR 0.68, 0.31, and 0.14 groups quadratically increased the level of serum glucose at 3 and 5 h post-prandium (*p* < 0.01) and linearly increased at 4 h post-prandium (*p* < 0.05). Furthermore, the variation in insulin level did not respond correspondently to the postprandial blood glucose levels. The fasting insulin concentration quadratically increased in AR 2.90, 1.46, 0.68, and 0.31 groups, compared with piglets consuming the AR 0.14 diet (*p* < 0.01). Compared with the AR 0.31 and 0.14 groups, the insulin level (*p* < 0.01) at 1 h post-prandium was quadratically increased in AR 2.90, 1.46, and 0.68 groups. However, piglets in AR 0.14 group quadratically increased their insulin concentrations at 2, 3, 4, and 5 h post-prandium (*p* < 0.01). Moreover, the serum insulin levels in the five dietary treatment groups were increased to the peak at 2 h post-pranidum, and then decreased gradually over time. Overall, the serum insulin concentration in the AR 0.14 group showed greater changes than that of AR 2.90 group at 2, 3, 4, and 5 h post-prandium (*p* < 0.05).

### 3.3. Nutrient Digestibility

Piglets fed the high amylopectin diet (AR 0.14) significantly increased (*p* < 0.001) the digestibility of DM, EE, GE, CP, and ash compared with the other four groups (Table 7). Piglets that consumed the AR 0.68 diet had decreased (*p* < 0.05) the digestibility of DM, EE, GE, CP, and ash compared with piglets consuming the high amylose diet (AR 2.90). 

### 3.4. Concentration of Cecal Digesta VFA and pH Value

VFA concentrations in the cecal digesta are presented in Table 8. It was shown that diets with different AR did not affect the total concentration of VFA in the cecum. However, increasing dietary amylose quadratically increased (*p* < 0.05) the butyric acid concentration. Piglets that consumed the high amylose diet (AR 2.90) tend to have greater cecal concentration of acetic acid (*p* = 0.070) and propionic acid (*p* = 0.087, linear effect) than piglets consuming other four diets. In addition, increasing dietary amylopectin content linearly increased the cecal digesta pH value (*p* < 0.05) (Table 8).

### 3.5. Intestinal Gene Expression

**Figure 1:**
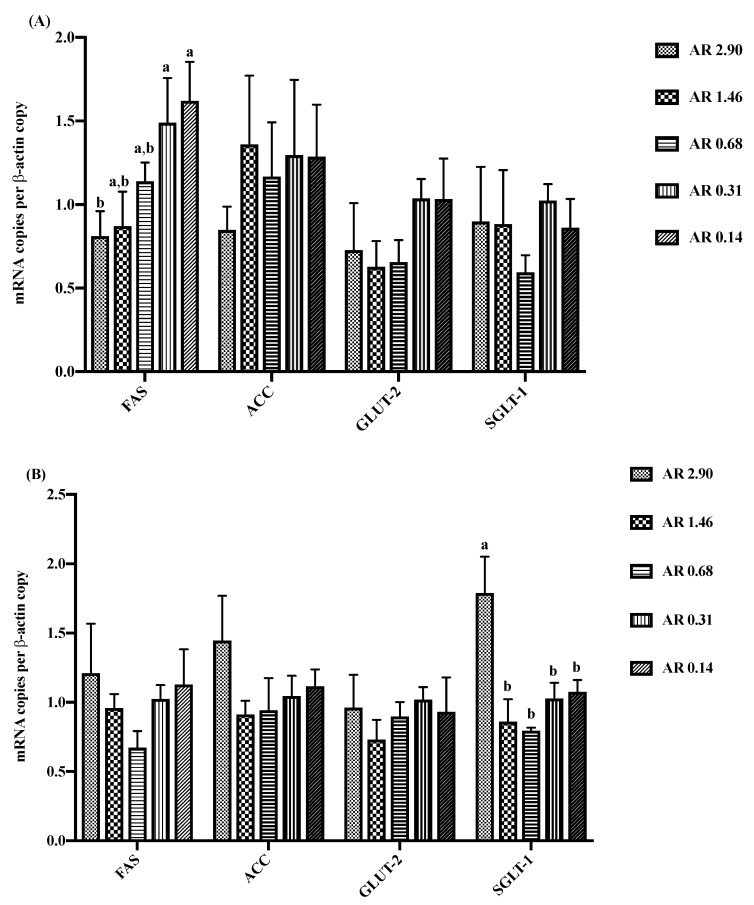
Effects of dietary starch structure on intestinal gene expression in the duodenum (**A**) and jejunum (**B**). *FAS*, fatty acid synthase; *ACC*, acetyl CoA carboxylase; *GLUT-2*, glucose transporter type 2; *SGLT-1*, sodium-dependent glucose transporter 1. Values are means, with standard errors represented by vertical bars (*n* = 6). ^a, b^ Mean values with unlike letters were significantly different within a cluster of bars, not across the clusters of bars (*p* < 0.05).

The effects of DAR on intestinal gene expression are presented in Figure 1. Quantitative RT-PCR assays were designed for four genes expressed in duodenum and jejunum. The genes were selected based on their involvement in lipid metabolism and glucose transport in duodenal and jejunal mucosa. The expression of gene *FAS* tended to increase (*p* = 0.083) in the duodenum when piglets were fed with low AR (AR 0.14) diet. However, the expression of *SGLT1* in the jejunum increased (*p* < 0.01) in high AR (AR 2.90) diet.

### 3.6. Intestinal Microbiota

Cecal microflora data are shown in Table 9. The populations of *Bacillus*, *Bifidobacterium*, and *Lactobacillus* showed no differences (*p* > 0.05) in piglets fed diets with different AR. However, piglets fed the high amylopectin diet (AR 0.14) had quadratically increased (*p* < 0.001) total bacteria populations compared with piglets consuming the other 4 diets. Increasing dietary amylose had a linear tend to decrease (*p* = 0.075) *Escherichia coli* populations compared with piglets consuming the low AR diets.

## 4. Discussion

Starch is the main carbohydrate available for humans and monogastric animals [23]. It has been proposed that starch can be classified as rapidly digestible starch (RDS), slowly digestible starch (SDS), and RS according to digestion in the anterior of small intestine [24,25]. Amylose is difficult to hydrolyze, while amylopectin can be easily digested. Therefore, the ratio of amylose to amylopectin may affect metabolic and physiological responses [26]. In the present study, we evaluated the effects of diets with five different AR on the growth performance; fasting; and postprandial levels of glucose and insulin, nutrient digestibility, and microbial profiles in weaned piglets. Purified starch sources were used to minimize the confounding effects of intrinsic starch-associated compounds, such as fat, fiber, and protein.

Previous studies showed that young pigs fed with a high amylose diet significantly decreased growth performance and feed efficiency [10]. In the present study, the growth performance of piglets was not affected by DAR. This is probably due to the difference in amylose content, as an extremely high amylose content (more than 75%) was used in the current study [10]. However, our results are consistent with previous studies performed in weaned and growing–finishing pigs [11,12]. 

The present results showed that increasing dietary amylopectin content was associated with increased nutrient digestibility, which increased the postprandial glucose–insulin response. In addition, previous studies have demonstrated that ingestion of RDS leads to rapid increase in blood glucose and triggers insulin secretion from β-cells of the endocrine pancreas [23,27]. Typically, insulin secretion maintains blood glucose homeostasis [28]. This study showed that there is a clear correlation between the starch digestibility and insulin response to diet with different AR. We found that glucose concentration at 3 h post-prandium in piglets in the high AR (AR 2.90 and 1.46) groups were lower than those in the low AR group. In contrast, the serum insulin levels in piglets in the low AR (AR 0.31) group showed greater changes from peak to 5 h post-prandium compared with high AR groups, and these fluctuations were regular. These results suggested that diet with high AR had a stable glucose–insulin response. Similar insulinemia responses were observed in both pigs [8] and humans [29] when a fed a high amylose starch diet. In addition, long-term ingestion of diet with low AR may produce a prolonged increase in serum insulin concentration with decrease insulin sensitivity. Previous studies have indicated that insulin signaling can stimulate rapidly glucose transport, as well as fatty acid uptake, which is the aim of promoting fat synthesis [8]. Moreover, the improved utilization of glucose might result from the elevated insulin concentration, which is the most potent physiological anabolic agent, promoting the storage of lipids [30]. This hypothesis was verified by the measurements of the lipogenic gene expression produced in the duodenal mucosa. We found that the mRNA level of *FAS* in piglets of the low AR (AR 0.31 and 0.14) groups were higher than those of the high AR groups. The present results, however, agree well with previous findings showing that ingestion of a low AR diet significantly elevated the lipogenic gene expression in growing–finishing pigs [31] and other animals [32,33].

To further understand the effects of DAR on intestinal health, cecal digesta samples were collected to measure SCFAs and microbes. We found that diets with high levels of amylose linearly decreased the digesta pH value, which was probably due to microbial fermentation. It is a well-known fact that SCFAs are the major products of indigestible starch fermentation in the large intestine and only a few SCFAs are excreted in feces [34,35,36,37]. In the present study, we found that increases in the amylose ratio significantly elevated the butyrate concentration in the cecum, which indicated a beneficial role of a high amylose diet in maintaining intestinal health, as the butyrate was reported to serve as a critical energy source for enterocytes and it can improve the intestinal morphology and barrier function [38]. To our astonishment, no significant differences in the abundance of beneficial bacteria, such as *Bacillus*, *Lactobacillus*, and *Bifidobacterium*, were observed. However, the result is consistent with the previous report showing that ingestion of a high amylose diet did not increase several beneficial bacteria populations, such as *Bifidobacterium spp.* and *Firmicutes*, in the cecum of growing pigs [39]. Additionally, diet containing raw potato starch (high AR) had no effect on the lactic acid bacterial counts or the ratio of lactic acid bacteria to *E. coli* in weaned pigs [40].

## 5. Conclusions

In conclusion, the present results suggested that the metabolic responses of weaned piglets fed with different DAR may vary widely depending on their composition, and ingestion of a low AR diet leads to a stronger insulinemic response and up-regulation the expression of lipogenic genes. In contrast, ingestion of a high AR diet is helpful for maintaining the intestinal microbiota and health.

## Figures and Tables

**Table 1:** Ingredients and chemical composition of experimental diets (as-fed basis).

Item	Content
Ingredients (%)	
Extruded soya bean	6.50
Soybean meal, dehulled	14.00
Fish meal (62.5%)	5.00
Whey powder	8.50
Blood plasma meal	3.25
Soy protein concentrate	7.50
Cellulose	1.20
Limestone	1.04
Dicalcium phosphate	0.13
Glucose	2.00
Salt	0.35
L-Lys·HCl (78%)	0.04
DL- **Methionine**	0.09
Chloride choline	0.15
Vitamin and minerals premix ^1^	0.25
Total	100.00
Calculated content (as DM)	
Dietary energy (Mcal/kg)	3.68
Crude protein (%)	19.37
Ca (%)	0.80
Available P (%)	0.36
Methionine+ cystiene (%)	0.72
Threonine (%)	0.82
Tryptophan (%)	2.05
Crude fiber (%)	2.05

^1^ Supplied (per kg diet): Vitamin A, 6000 IU; Vitamin D_3_, 400 IU; Vitamin E, 10 IU; Vitamin K_3_, 2 mg; Vitamin B_1_, 0.8 mg; Vitamin B_2_, 6.4 mg; Vitamin B_6_, 2.4 mg; Vitamin B_12_, 12 µg; folic acid, 0.2 mg; nicotinic acid, 14 mg; *D*-pantothenic acid, 10 mg. Supplied (per kg diet): Fe as FeSO_4_·7H_2_O, 100 mg; Mn as MnSO_4_·7H_2_O, 4 mg; Zn as ZnSO_4_, 80 mg; Cu as CuSO4·5H_2_O, 100 mg; Se as Na_2_SeO_3_, 0.35 mg; and I as KI, 0.3 mg.

**Table 2:** Primer sequences of genes selected for analysis by real-time PCR.

Gene	Primer Sequence (5’-3’)	Accession No.	Temp (°C)
*FAS*	Forward: GGACCTGGTGATGAACGTCT	EF589048	65.0
	Reverse: CGGAAGTTGAGGGAGGTGTA		
*ACC*	Forward: ATGTTTCGGCAGTCCCTGAT	EF618729	59.7
	Reverse: TGTGGACCAGCTGACCTTGA		
*GLUT2*	Forward: TGGAATCAGCCAACCTGTTT	NM_001097417.1	59.7
	Reverse: ACAAGTCCCACCGACATGA		
*SGLT1*	Forward: AGAAGGGCCCCAAAATGACC	NM_001164021.1	65.0
	Reverse: TGTTCACTACTGTCCGCCAC		
*β-Actin*	Forward: TCTGGCACCACACCTTCT	XM_003124280.3	60.0
	Reverse: TGATCTGGGTCATCTTCTCAC		

*FAS* = fatty acid synthase; *ACC* = acetyl CoA carboxylase; *GLUT2* = glucose transporter type 2; *SGLT1* = sodium-dependent glucose transporter 1.

**Table 3:** Primer and probe sequences used for real-time quantitative PCR analysis of selected microbial populations in cecal digesta samples.

Item	Primer/Probe Name and Sequence (5’-3’)	Size (bp)	Annealing Temperature (°C)
*Bifidobacterium*	SQ-F, CGCGTCCGGTGTGAAAG	121	55.0
	SQ-R, CTTCCCGATATCTACACATTCCA		
	SQ-P, (FMA) ATTCCACCGTTACACCGGGAA(BHQ-1)		
*Lactobacillus*	RS-F, GAGGCAGCAGTAGGGAATCTTC	126	53.0
	RS-R, CAACAGTTACTCTGACACCCGTTCTTC		
	RS-P, (FMA)AAGAAGGGTTTCGGCTCGTAAAACTCTGTT(BHQ-1)		
*Bacillus*	YB-F, GCAACGAGCGCAACCCTTGA	92	53.0
	YB-R, TCATCCCCACCTTCCTCCGGT		
	YB-P, (FMA)CGGTTTGTCACCGGCAGTCACCT(BHQ-1)		
*Escherichia coli*	DC-F, CATGCCGCGTGTATGAAGAA	96	55.0
	DC-R, CGGGTAACGTCAATGAGCAAA		
	DC-P, (FMA)AGGTATTAACTTTACTCCCTTCCTC(BHQ-1)		
Total bacteria	Eub338F, ACTCCTACGGGAGGCAGCAG		
	Eub518R, ATTACCGCGGCTGCTGG	200	61.5

**Table 4:** Effects of dietary starch structure on the growth performance in weaned piglets (mean values with their standard errors).

Item	Treatments					*p*-Value		
	AR 2.90 ^1^	AR 1.46	AR 0.68	AR 0.31	AR 0.14	ANOVA	Linear	Quadratic
ADFI, g/d ^2^	522.46 ± 15.86	541.78 ± 22.71	539.11 ± 7.18	531.90 ± 16.99	533.96 ± 14.04	0.949	0.810	0.812
ADG, g/d ^3^	302.92 ± 15.86	314.76 ± 14.07	304.97 ± 9.69	296.85 ± 8.51	300.22 ± 12.26	0.876	0.540	0.786
F/G, g/d ^4^	1.73 ± 0.04	1.73 ± 0.06	1.78 ± 0.07	1.79 ± 0.04	1.79 ± 0.06	0.851	0.288	0.564

^1.^ AR = amylose/amylopectin ratio; ^2^ ADFI = average daily feed intake; ^3^ ADG = average daily gain; ^4^ F/G = the ratio of average daily feed intake to average daily gain (*n* = 18).

**Table 5:** Effects of dietary starch structure on serum glucose in weaned piglets (mean values with their standard errors) ^1^.

Item	Treatments					*p*-Value		
	AR 2.90 ^2^	AR 1.46	AR 0.68	AR 0.31	AR 0.14	ANOVA	Linear	Quadratic
GLU F ^3^ (mmol/L)	5.95 ± 0.52	6.11 ± 0.35	6.33 ± 0.33	5.70 ± 0.49	5.76 ± 0.64	0.882	0.589	0.727
GLU 1h ^4^ (mmol/L)	8.66 ± 0.57	8.42 ± 1.07	8.57 ± 0.73	8.32 ± 0.67	8.70 ± 1.20	0.998	0.332	0.629
GLU 2h (mmol/L)	6.84 ± 0.45	7.48 ± 0.52	7.45 ± 0.65	7.63 ± 0.30	7.82 ± 0.72	0.769	0.208	0.432
GLU 3h (mmol/L)	6.89 ± 0.35 ^b^	6.59 ± 0.26 ^b^	8.76 ± 0.51 ^a^	8.88 ± 0.45 ^a^	8.34 ± 0.37 ^a^	0.001	0.002	0.004
GLU 4h (mmol/L)	6.05 ± 0.21	5.92 ± 0.16	6.90 ± 0.37	6.55 ± 0.36	7.02 ± 0.54	0.103	0.022	0.077
GLU 5h (mmol/L)	5.60 ± 0.11 ^b^	6.06 ± 0.18 ^b^	6.97 ± 0.43 ^a^	5.70 ± 0.25 ^b^	5.59 ± 0.31 ^b^	0.008	0.723	0.024

^1 a,b^ Mean values within a row with unlike superscript letters were significantly different (*p* < 0.05); ^2^ AR = amylose/amylopectin ratio; ^3^ GLU F = serum fasting glucose concentration; ^4^ GLU 1h-GLU 5h: once per hour collected postprandial blood samples (*n* = 6).

**Table 6:** Effects of dietary starch structure on serum insulin in weaned piglets (mean values with their standard errors) ^1^.

Item	Treatments					*p*-Value		
	AR 2.90^2^	AR 1.46	AR 0.68	AR 0.31	AR 0.14	ANOVA	Linear	Quadratic
INS F ^3^ (mIU/L)	57.20 ± 4.96 ^a^	61.72 ± 3.98 ^a^	65.00 ± 1.95 ^a^	54.41 ± 3.07 ^a^	37.69 ± 3.61 ^b^	<0.001	0.003	<0.001
INS 1h ^4^ (mIU/L)	77.73 ± 4.01 ^b^	84.15 ± 1.43 ^ab^	89.82 ± 2.29 ^a^	66.25 ± 2.97 ^c^	69.27 ± 1.23 ^c^	<0.001	0.009	0.001
INS 2h (mIU/L)	91.40 ± 6.19 ^b^	91.57 ± 2.12 ^b^	90.96 ± 2.32 ^b^	112.26 ± 1.88 ^a^	108.45 ± 3.53 ^a^	<0.001	<0.001	0.001
INS 3h (mIU/L)	54.62 ± 2.42 ^b^	64.72 ± 5.93 ^b^	66.49 ± 1.73 ^b^	100.59 ± 6.08 ^a^	104.73 ± 4.06 ^a^	<0.001	<0.001	<0.001
INS 4h (mIU/L)	90.98 ± 3.12 ^ab^	83.26 ± 4.09 ^b^	85.29 ± 2.91 ^b^	80.31 ± 3.27 ^b^	100.33 ± 6.64 ^a^	0.020	0.385	0.015
INS 5h (mIU/L)	53.55 ± 3.83 ^b^	47.21 ± 0.46 ^b^	47.70 ± 5.59 ^b^	59.01 ± 3.03 ^ab^	69.83 ± 8.27 ^a^	0.026	0.092	0.022

^1 a,b^ Mean values within a row with unlike superscript letters were significantly different (*p* < 0.05); ^2^ AR = amylose/amylopectin ratio; ^3^ INS F = serum fasting insulin concentration; ^4^ INS 1h-INS 5h: once per hour collected postprandial blood samples (*n* = 6).

**Table 7:** Effects of dietary starch structure on apparent total tract digestibility in weaned piglets (mean values with their standard errors) ^1^.

Items	Treatments					*p*-Value
	AR 2.90 ^2^	AR 1.46	AR 0.68	AR 0.31	AR 0.14	
Dry matter	85.39 ± 0.58 ^c^	83.98 ± 0.41^c^	70.36 ± 3.56 ^d^	87.15 ± 0.44 ^bc^	93.14 ± 0.93 ^a^	<0.001
Ether extract	90.84 ± 0.17 ^a^	78.11 ± 1.20 ^b^	64.02 ± 7.57 ^c^	80.31 ± 0.64 ^b^	92.56 ± 0.24 ^a^	<0.001
Gross energy	83.26 ± 0.67 ^b^	81.06 ± 0.56 ^b^	64.99 ± 4.95 ^c^	85.52 ± 0.40 ^b^	92.71 ± 0.34 ^a^	<0.001
Crude protein	73.43 ± 0.88 ^c^	64.19 ± 0.79 ^d^	37.47 ± 3.02 ^e^	78.89 ± 0.47 ^b^	88.19 ± 0.45 ^a^	<0.001
Crude Ash	60.77 ± 1.51 ^bc^	49.31 ± 1.94 ^cd^	38.36 ± 6.09 ^d^	64.26 ± 5.24 ^ab^	77.09 ± 0.81 ^a^	<0.001

^1 a,b,c,d,e^ Mean values within a row with unlike superscript letters were significantly different (*p* < 0.05); ^2^ AR = amylose/amylopectin ratio (*n* = 6).

**Table 8:** Effects of dietary starch structure on the intestinal microbial metabolites (μmol/g of wet digesta) and pH value in the cecum in weaned piglets (mean values with their standard errors) ^1^.

Item	Treatments					*p*-Value		
	AR 2.90^2^	AR 1.46	AR 0.68	AR 0.31	AR 0.14	ANOVA	Linear	Quadratic
Acetic acid	58.26 ± 3.11 ^ab^	51.34 ± 6.20 ^b^	50.89 ± 3.65 ^b^	66.70 ± 4.12 ^a^	61.11 ± 3.51 ^ab^	0.070	0.158	0.206
Propionic acid	31.05 ± 3.98	25.07 ± 2.93	24.20 ± 4.30	22.55 ± 1.06	23.65 ± 2.48	0.375	0.087	0.122
Butyric acid	22.36 ± 3.48 ^a^	20.62 ± 3.92 ^a^	20.05 ± 2.20 ^a^	16.60 ± 1.44 ^ab^	9.39 ± 1.78 ^b^	0.020	0.002	0.003
Total volatile fatty acid	111.67 ± 2.56	97.03 ± 9.02	95.13 ± 8.01	105.85 ± 5.91	94.15 ± 7.52	0.335	0.248	0.409
pH value	5.39 ± 0.09 ^b^	5.58 ± 0.10 ^ab^	5.46 ± 0.08 ^ab^	5.63 ± 0.14 ^ab^	5.79 ± 0.17 ^a^	0.203	0.033	0.093

^1 a,b^ Mean values within a row with unlike superscript letters were significantly different (*p* < 0.05); ^2^ AR = amylose/amylopectin ratio (*n* = 6).

**Table 9:** Effects of dietary starch structure on the cecal bacterial community in weaned piglets (log (copies·g −1)) ^1^ (mean values with their standard errors).

Item	Treatments					*p*-Value		
	AR 2.90 ^2^	AR 1.46	AR 0.68	AR 0.31	AR 0.14	ANOVA	Linear	Quadratic
Total bacteria	12.16 ± 1.14 ^c^	12.22 ± 0.85 ^c^	12.20 ± 0.77 ^c^	15.45 ± 0.33 ^b^	17.98 ± 0.66 ^a^	<0.001	<0.001	<0.001
*Bacillus*	8.75 ± 0.15	9.06 ± 0.25	8.93 ± 0.14	8.69 ± 0.27	8.85 ± 0.21	0.740	0.794	0.843
*Lactobacillus*	7.49 ± 0.20	7.56 ± 0.25	7.78 ± 0.26	7.25 ± 0.17	7.57 ± 0.22	0.583	0.828	0.936
*Bifidobacterium*	5.13 ± 0.14	5.16 ± 0.20	5.03 ± 0.14	4.99 ± 0.22	5.25 ± 0.27	0.891	0.909	0.748
*Escherichia coli*	8.14 ± 0.17	7.85 ± 0.40	8.18 ± 0.42	8.37 ± 0.40	8.82 ± 0.20	0.344	0.075	0.113

^1 a,b^ Mean values within a row with unlike superscript letters were significantly different (*p* < 0.05); ^2^ AR = amylose/amylopectin ratio (*n* = 6).

## Supplementary Materials

The following are available online at https://www.mdpi.com/2076-2615/10/3/543/s1, Figure S1: Effects of dietary starch structure on serum glucose and insulin concentrations.
