# Supplementary material for: Effects of Dietary Starch Structure on Growth Performance, Serum Glucose–Insulin Response, and Intestinal Health in Weaned Piglets
Source: Animals (Basel). 2020 Mar 24;10(3):543. doi: 10.3390/ani10030543 (PMC7142567; doi:10.3390/ani10030543)
Supplement: Supplementary file 1 [file animals-10-00543-s001.pdf]

Additional file:

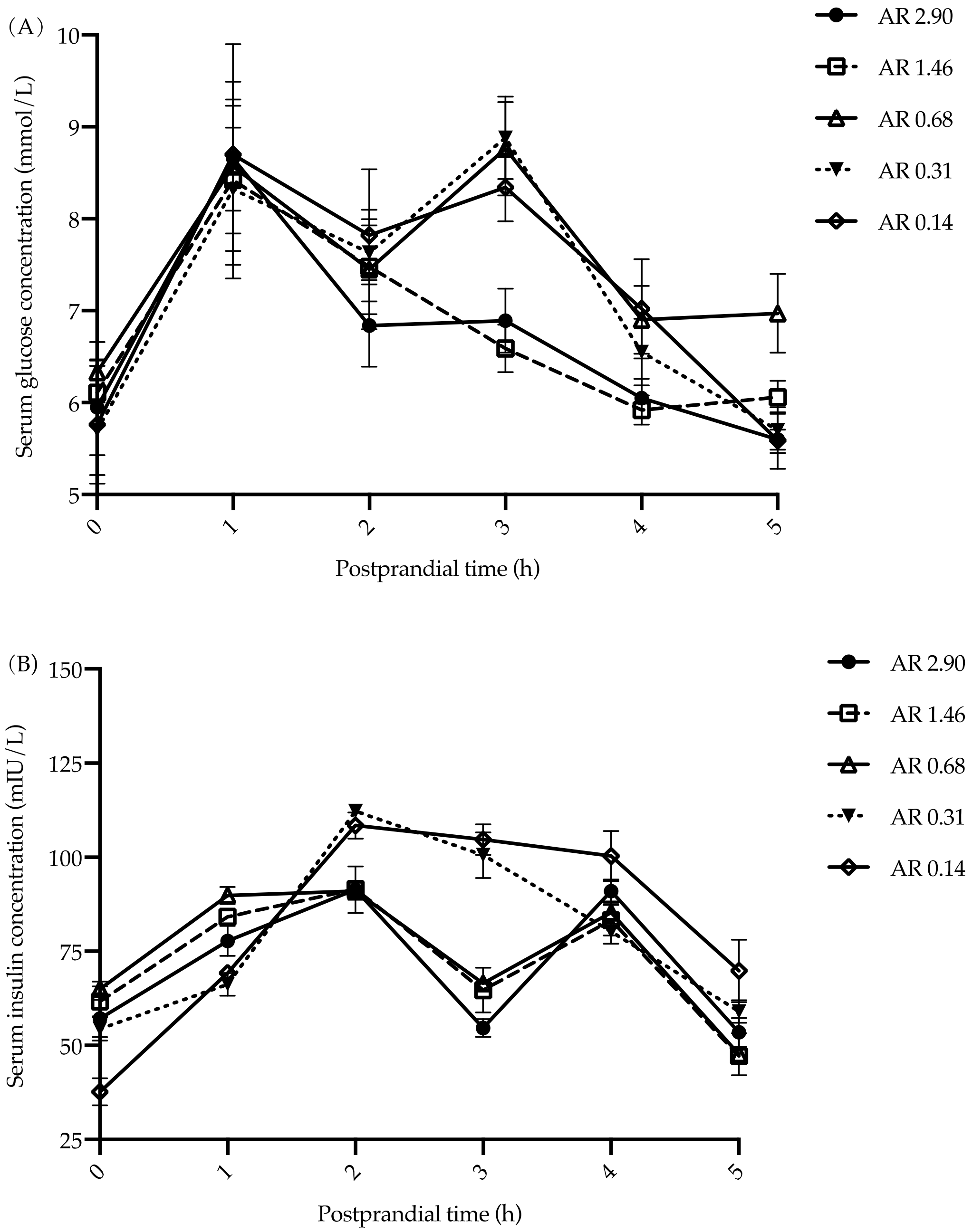

Figure S1. Effects of dietary starch structure on serum glucose concentration (A) and insulin concentration (B). Samples were collected during the first 5 h after feeding. Values are means (n =6). Significant refer to Tables 5 and 6, respectively.
